# Orbital Compartment Syndrome Following Mechanical Fall

**DOI:** 10.5811/cpcem.2018.4.37810

**Published:** 2018-06-12

**Authors:** Arielle E. Schwitkis, Talia L. Pollack, Sam S. Torbati

**Affiliations:** Cedars-Sinai Medical Center, Department of Emergency Medicine, Los Angeles, California

## CASE PRESENTATION

An 80-year-old woman with a history of hypertension presented to the emergency department (ED) with blunt facial trauma including a four-centimeter laceration of the right upper eyelid sustained during a ground-level mechanical fall. Upon arrival to the ED, she was confused, repetitive, and amnesic to events surrounding the fall. Computed tomography (CT) of the brain and orbits was rapidly obtained, and upon return from CT she reported new visual loss of the right eye with the ability to see only light. On exam, her globe was noted to be increasingly firm, full to palpation, and swollen shut. Physical examination also revealed new ophthalmoplegia, proptosis, subconjunctival hemorrhage, and afferent pupillary defect. Intraocular pressure (IOP) measured 50 mmHg in the right eye and 12 mm Hg in the left eye. CT demonstrated a hematoma within the right orbit impinging on orbital contents, confirming the diagnosis of orbital compartment syndrome (OCS). An emergent bedside lateral canthotomy and cantholysis (LCC) was performed by the emergency physician with reduction of her IOP and restoration of vision.

## DISCUSSION

OCS is a rare complication of increased pressure within the confined orbital space. It may be caused by retro-orbital hematoma following blunt or penetrating trauma to the orbit. The pressure exerted by the hematoma reduces perfusion resulting in ischemia-induced vision loss, which may develop over minutes to hours.^1^–^3^ OCS may present with ocular pain, diplopia, or vision loss.^4^,^5^ OCS is a clinical diagnosis with physical examination findings that may include ophthalmoplegia, proptosis, subconjunctival hemorrhage, and afferent pupillary defect.^1^,^4^,^5^ Diagnostic criteria include a constellation of the aforementioned signs and symptoms associated with an IOP of 30 mmHg or higher.^2^,^6^ LCC, the primary treatment for OCS, is a relatively simple procedure ideally performed within 60–120 minutes of symptom onset to prevent permanent vision loss.^1^,^3^,^4^,^7^

**Image 1:**
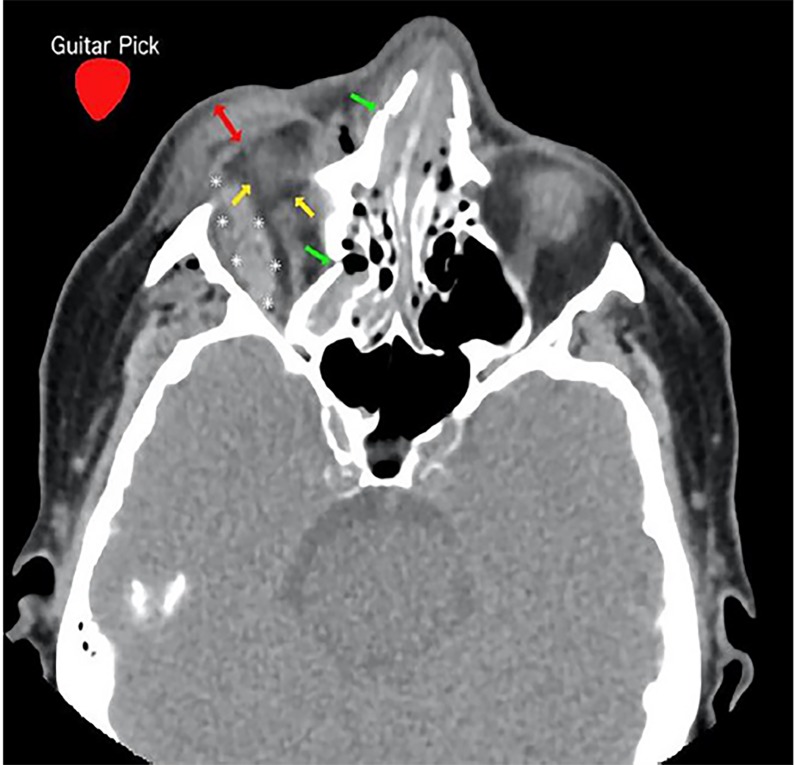
Computed tomography of facial bones without contrast, axial view, demonstrating retro-orbital hematoma (asterisks); tenting of posterior sclera (also known as “guitar pick” sign) (yellow arrows); eyelid edema (red two-way arrow); bony fractures (green arrows), and superimposed image of a guitar pick (red guitar pick with white text). The hematoma within the orbit is impinging on the orbital contents, pushing it medially and slightly superiorly.

**Image 2:**
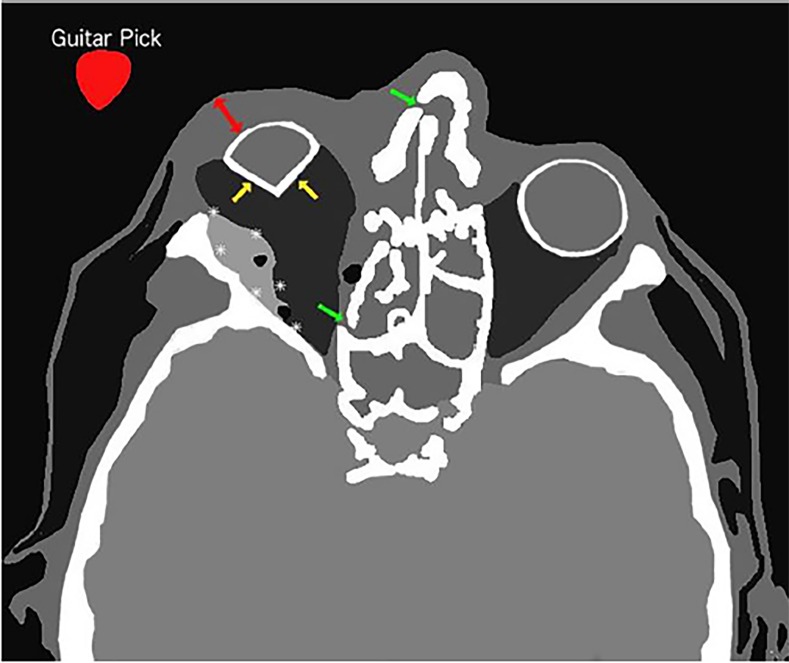
Artistic rendering of computed tomography of facial bones without contrast, axial view, demonstrating retro-orbital hematoma (asterisks), tenting of posterior sclera (also known as “guitar pick” sign) (yellow arrows), eyelid edema (red two-way arrow), bony fractures (green arrows), and superimposed image of a guitar pick (red guitar pick with white text).

Diagnosis of OCS can be challenging as the patient’s examination may be limited by altered mental status; vision loss may be masked by inability to open edematous eyelids; and orbital pain may be explained by bony and soft tissue injury.^7^ CT findings concerning for OCS include tenting of the posterior sclera – otherwise known as “guitar pick” sign – caused by intraocular mass (Images 1 and 2).^8^ Although CT findings of retro-orbital hematoma should raise suspicion for OCS, serial evaluations are essential for detecting OCS in evolution.

Documented patient informed consent and/or Institutional Review Board approval has been obtained and filed for publication of this case report.

**CPC-EM Capsule**

What do we already know about this clinical entity?

Orbital compartment syndrome is a rare complication of increased pressure within the confined orbital space that may lead to permanent blindness if not treated in a timely fashion.

What is the major impact of the image(s)?

The “guitar pick” sign is a rarely reported radiographic sign that should alert physicians of the possibility of a syndrome requiring immediate intervention.

How might this improve emergency medicine practice?

Emergency physicians will be able to better recognize patients at risk for orbital compartment syndrome following blunt facial trauma.
